# The Effects of a Multicomponent Group Exercise Program on Physical Performance and Sleep Quality in Older Adults in Taiwan

**DOI:** 10.1177/10105395261469631

**Published:** 2026-07-25

**Authors:** Yi-Ling Tseng, Kuei-Min Chen, Min-Huan Wu, Jeng Liu, Ying-Chi Liang, Wendy Moyle

**Affiliations:** 1 College of Nursing, Kaohsiung Medical University, Taiwan; 2 Tunghai University, Taichung, Taiwan; 3 Center for Long-term Care Research, Kaohsiung Medical University, Taiwan; 4 Department of Medical Research, Kaohsiung Medical University Hospital, Taiwan; 5 Department of Sociology, Tunghai University, Taichung, Taiwan; 6 Institute of Community Health Care, College of Nursing, National Yang Ming Chiao Tung University, Taipei, Taiwan; 7 School of Nursing and Midwifery, Griffith University, Brisbane, QLD, Australia

**Keywords:** exercise, older adult, health promotion, muscle strength, physical performance, sleep quality

## Abstract

The aging population necessitates proactive health interventions. This study evaluated the impact of a multicomponent group exercise program on physical performance and sleep quality in community-dwelling older adults using a quasi-experimental control-group design. The study was conducted in 2024 at a university-affiliated senior fitness club in central Taiwan, involving 80 participants from community care sites. The intervention group completed an 8-week multicomponent group exercise program, while the control group maintained their community activities. Physical performance and sleep quality were assessed before and after. After the intervention, the intervention group showed a significant difference in gait speed, upper limb muscle strength, sleep quality, and heart rate recovery. Between groups, significant differences were observed in arm curls, grip strength, and sleep quality in the posttest. An 8-week multicomponent group exercise program positively impacted muscle strength and sleep quality in older adults, supporting its implementation in community.

Trial Registration: ClinicalTrials.gov, https://clinicaltrials.gov, NCT07598123.

## What We Already Know

- The exercise intervention has been shown to improve mobility, balance, and quality of life in community-dwelling older adults.
- Research on combining structured group exercise programs with smart fitness equipment for older adults as an intervention remains insufficient.
- Incorporating facilitators such as peer support, appropriate exercise equipment, and motivation into the design of exercise interventions can enhance participation among older adults.

## What This Article Adds

- An 8-week multicomponent group exercise program had a positive effect on limb muscular strength and endurance.
- Promoting the multicomponent group-based exercise programs may offer potential health benefits for older adults by improving sleep quality as a nonpharmacological treatment strategy.
- Integrating senior-friendly and smart fitness equipment into the exercise programs for older adults can strengthen the monitoring of training dose, personalized interventions, and provide a new strategy for policy-driven technology-assisted health promotion.

## Introduction

The World Health Organization (WHO) estimates that by 2030, there will be between 1.1 and 1.4 billion people worldwide who are 60 years of age or older.^1^ Early mobility assessments and exercise interventions can prevent frailty progression^2^ and lower the risk of sarcopenia, osteoporosis, falls, and chronic conditions such as hypertension, diabetes, obesity, insomnia, and depression.^3^ A longitudinal study reported that regular weekly physical activity reduced the risk of all-cause mortality by 40%.^4^ While another study found that physical activity was positively associated with healthy aging and lowered the risk of rapid decline in older adults.^5^

Taiwan’s healthy aging policy focuses on health promotion and prevention. To encourage fitness, the Ministry of Health and Welfare launched the Senior Fitness Club Pilot Program in 2020 for persons aged 50 years and older. Each club operates at least 2 days a week, offering a minimum of 2 hours of guided multimodal training per session. By linking local health centers, schools, government agencies, and community hubs, the program delivers structured exercise, Integrated Care for Older People (ICOPE) assessments by certified instructors, tailored exercise programs based on assessment results, performance monitoring, and participation in health-promotion activities.^6^

The American College of Sports Medicine (ACSM) provides evidence-based guidelines regarding exercise frequency, intensity, and duration for older adults. The ACSM recommends at least 150 minutes of moderate-intensity aerobic activity each week. Relative intensity refers to the level of effort required for an activity, as perceived by the individual performing it. It is measured on a 10-point scale of perceived exertion scale, with moderate intensity ranging from 5 to 6. Resistance training should be performed twice a week and include progressive weight training that targets 8 to 10 major muscle groups, with 8 to 12 repetitions per exercise. Flexibility exercises are also recommended twice weekly. Furthermore, intensity and duration should begin at low levels and gradually increase based on personal tolerance.^7^

Although structured exercise interventions for healthy, community-dwelling older adults have been shown to improve physical function and quality of life significantly,^8-11^ only a few controlled studies have evaluated the efficacy of multicomponent exercise training for sleep disturbances in older adults.^12^ This study addressed a gap in the literature regarding the health benefits of a program that combines structured group exercise with senior-friendly fitness equipment, especially their impact on sleep quality. Therefore, this study applied the evidence-based exercise guidelines for older adults provided by ACSM when designing a structured, multicomponent group exercise program and evaluated its effects on physical performance and sleep quality among older adults.

## Methods

This study followed a quasi-experimental pretest/posttest control group design with convenience sampling. The Institutional Review Board of Chung Shan Medical University Hospital (approval # CS1-24033) approved the study. The study was retrospectively registered in the ClinicalTrials. gov registry (registration no. NCT07598123). The study procedures, outcome measures, and ethical approval were completed prior to participant recruitment. All eligible participants received complete study information, and written informed consent was obtained. No identifying information was kept on the questionnaire, and identifying information was kept in a separate file.

### Participants

Four community care sites near the university-affiliated senior fitness club in central Taiwan were selected using convenience sampling. Participants were community-dwelling older adults who regularly attended these community care sites, which offered health-promotion programs and congregated meal services. Recruitment and study briefings were conducted on-site. A total of 127 participants from four community care sites were involved in the recruitment and eligibility screening process. Inclusion criteria were age ≥ 65 years, conscious, and able to communicate in Mandarin or Taiwanese. Exclusion criteria included contraindications to exercise (such as recent myocardial infarction, acute cardiovascular events or infections, or symptoms of renal disease), use of ambulatory aids, or engagement in regular physical activity exceeding 150 minutes per week. The Revised Physical Activity Readiness Questionnaire assessed exercise-related risks.^13,14^ Eligible participants were screened for exercise risks after consenting to participate in this study. Only those who answered “no” to any question on this scale, indicating lower exercise-related risks, can enroll in the intervention. The two community care sites that first reached recruitment targets were assigned to the intervention group, and the remaining two formed the control group. The research staff explained the study purpose and content of the questionnaire to 127 subjects, and 80 participants joined the study after completing the consent forms. The enrollment and allocation process is shown in the Supplemental Materials.

Participants in this study were older adults without a history of consistent strength training. The effect size was defined as 0.6 for untrained individuals, based on Rhea,^15^ with an α level of .05 and a power of 0.8. According to Cohen’s^16^ criteria, an effect size between 0.5 and 0.8 is considered medium. Power analysis indicated that 36 participants per group (N = 72) were required to detect a medium effect. Accounting for attrition, the target sample size was adjusted to 80, with 40 participants assigned to each group.

### Data Collection and Intervention

The study was conducted between May and October 2024. Pretest assessments and posttest evaluations were collected at four community care sites. The intervention took place at a university-affiliated senior fitness club in central Taiwan. The participant’s flow is shown in the Supplemental Materials.

The exercise program at the university-affiliated senior fitness club was collaboratively designed by experts in geriatric care, sports medicine, and certified fitness coaches, incorporating aerobic, resistance, and balance exercises tailored for older adults. A certified coach and four trained university students assisted on-site and followed a standardized exercise program schedule. They had previously received training in equipment use and basic life support, and emergency equipment was available at the club. Coaches briefed participants on procedures and safety precautions before each session, while trained assessors measured blood pressure.

After consulting with leaders and participants from the two participating settings about the shuttle bus schedule, the intervention was revised as an 8-week, 100-minute weekly group session. In the 100-minute weekly group session, participants began with 10 minutes of warm-up activities, involving extremity stretching and flexibility exercises. This was followed by 45 minutes of aerobic exercises, which included 15 minutes of whole-body vibration training and 30 minutes on a treadmill or stationary bicycle. After a 5-minute break, participants performed 35 minutes of resistance exercises using isokinetic training devices, and the session concluded with 5 minutes of cool-down exercises through slow walking (Details of the intervention content is as shown in Supplemental Materials).

During sessions, participants wore smartwatches to record their heart rates and were assigned serial numbers. The collected data were used to monitor and calculate the participants’ maximal heart rate (HRmax). The aerobic exercise training utilized whole-body vibration, as well as treadmills or stationary bicycles equipped with smart panels that recorded speed, time, incline, total calories burned, and step frequency to maintain the appropriate intensity. The resistance exercise training utilized senior-friendly, hydraulic-based isokinetic training machines with smart panels that recorded personalized resistance levels to guide progressive overload.

Exercise intensity was monitored using the session rating of perceived exertion (sRPE)^17,18^ and HRmax.^19^ In line with ACSM recommendations, participants maintained a moderate intensity, targeting a maximum heart rate of 5 to 6 on the sRPE scale^7^ and achieving 60% of their HRmax. HRmax, the maximum number of beats per minute, was estimated using HRmax = 208 − (0.7 × age).^19^ The whole-body vibration used in this intervention was delivered through a vertical, side-alternating platform. Based on previous literature,^20,21^ the vibration frequency was set at 20 to 35 Hz, and the vibration magnitude at 2 to 5 mm. It started at a low vibration frequency and was gradually increased every 2 weeks.

During the 8-week intervention period, participants in the control group were instructed to only continue their regular community activities and not to receive any additional physical training. Researchers visited the community care sites every 4 weeks to monitor the compliance of participants in the control group.

### Outcomes and Measuring Instruments

The primary outcomes were physical performance and sleep quality, and the secondary outcome was the heart rate recovery (HRR). Demographic data were collected using a structured self-developed questionnaire. Physical performance and sleep quality were assessed before and after the intervention using seven physical assessments and the Chinese version of the Pittsburgh Sleep Quality Index (CPSQI), respectively.

Trained assessors evaluated physical performance using seven adapted measurements: the 4-meter Gait Speed Test; the Chair Stand Test (five repetitions with arms crossed); the 30-second Arm Curl Test (2.27 kg for women, 3.63 kg for men); the Grip Strength Test using a hand dynamometer; the Side-by-Side Stand Test; the Tandem Stand Test (timed up to 10 seconds); and the 2.44-meter Timed Walk Test (stand, walk around a cone, and return to the chair).

Sleep quality was assessed with the CPSQI,^22^ a validated translation of the Pittsburgh Sleep Quality Index.^23^ It includes nine self-reported items scored across seven domains: subjective sleep quality, latency, duration, habitual efficiency, sleep disturbances, use of sleep medication, and daytime dysfunction, all assessed over the past month. Scores range from 0 to 21, with scores greater than five indicating poor sleep quality. The CPSQI was validated with good internal consistency (Cronbach’s α = .82-.83) and acceptable test-retest reliability over a 14- to 21-day period (r = 0.85).

Heart rate response during treadmill training was used to calculate the HRR, which reflects cardiovascular health. HRR was defined as the difference between the heart rate immediately after exercise and the heart rate measured 2 minutes later. The HRR value greater than 22 beats per minute (bpm) was associated with better cardiovascular function and a lower risk of mortality.^24^

### Statistical Analysis

Data was analyzed using the Statistical Package for the Social Sciences (SPSS) statistical software for Windows (version 25.0, IBM SPSS Inc.). Statistical tests were performed on baseline characteristics and preintervention outcome variables between the two groups prior to outcome analysis to assess baseline comparability. Due to abnormal distributions, continuous and ordinal variables were analyzed using the Mann-Whitney U test, while categorical variables were analyzed using Fisher’s exact test. To evaluate the intervention effects, the Wilcoxon Signed-Rank test was used for within-group comparisons, and the Mann-Whitney U test for between-group differences in physical performance and sleep quality. Only participants who completed both preintervention and postintervention assessments were included in the per-protocol analysis. In addition, an intention-to-treat (ITT) analysis was conducted to account for missing posttest data. Missing postintervention data in the ITT analysis were imputed using the last observation carried forward (LOCF) method. When controlling for potential confounding factors identified at baseline (eg, age and gender) was necessary, a nonparametric analysis of covariance was used to adjust for these covariates and assess intervention effects. Statistical significance was set at *P* ≤ .05.

## Results

### Participant Demographics

Eighty participants were initially recruited. A total of 75 community-dwelling older adults completed the study. Four participants dropped out from the intervention group, and 1 from the control group due to COVID infection, surgical hospitalization, and lower attendance rate, resulting in 36 and 39 participants remaining, respectively. Attrition rate was 6.3%, and adherence rate was 94.8%. No adverse events were reported during the intervention. Most participants were women (84%), with a mean age of 77.14 years (range 65-88). Baseline characteristics for each group are summarized in Table 1. There were no significant differences between the groups at baseline, indicating that the two groups were comparable.

**Table 1.** The Baseline Characteristics of Participants.

Variables	Intervention group (IG)	Control group (CG)	Z	*P*-value
**Per-protocol analysis (IG: n = 36, CG: n = 39)**				
Age (years), median (IQR)	77 (66-88)	78 (65-88)	−1.184	.236^a^
Gender, n (%)			−0.777	.535^b^
Male	7 (19.4)	5 (12.8)		
Female	29 (80.6)	34 (87.2)		
Marital status, n (%)				1.000^b^
With regular partner	35 (97.2)	37 (94.9)		
Without regular partner	1 (2.8)	2 (5.1)		
Education level			−1.46	.144^a^
Primary school and below	15 (41.7)	22 (56.4)		
Junior school	6 (16.6)	7 (18)		
High school and above	15 (41.7)	10 (25.6)		
Residence, n (%)				.520^b^
Living alone	4 (11.1)	7 (18)		
Living with spouse/children	32 (88.9)	32 (82)		
Chronic disease history, n (%)			−0.111	.911^a^
No chronic diseases	4 (11.1)	1 (2.6)		
One chronic disease	12 (33.3)	15 (38.5)		
Two chronic diseases	8 (22.3)	13 (33.3)		
Three or more chronic diseases	12 (33.3)	10 (25.6)		
Perceived health status, n (%)			−0.395	.693^a^
Very dissatisfied	1 (2.8)	3 (7.7)		
Dissatisfied	20 (55.6)	17 (43.6)		
Satisfied	14 (38.8)	17 (43.6)		
Very satisfied	1 (2.8)	2 (5.1)		
**Intention-to-treat analysis (IG: N = 40, CG: N = 40)**				
Age (years), median (IQR)	77 (66-88)	78 (65-88)	−1.320	.187^a^
Gender, n (%)				.378^b^
Male	9 (22.5)	5 (12.5)		
Female	31 (77.5)	35 (87.5)		
Marital status, n (%)				1.000^b^
With regular partner	39 (97.5)	38 (95)		
Without regular partner	1 (2.5)	2 (5)		
Education level			−1.352	.176^a^
Primary school and below	17 (42.5)	22 (55.0)		
Junior school	7 (17.5)	8 (20.0)		
High school and above	16 (40.0)	10 (25.0)		
Residence, n (%)				.518^b^
Living alone	4 (10)	7 (17.5)		
Living with spouse/children	36 (90)	33 (82.5)		
Chronic disease history, n (%)			−0.202	.840^a^
No chronic diseases	4 (10.0)	2 (5.0)		
One chronic disease	12 (30.0)	15 (37.5)		
Two chronic diseases	12 (30.0)	13 (32.5)		
Three or more chronic diseases	12 (30.0)	10 (25.0)		
Perceived health status, n (%)			−0.644	.520^a^
Very dissatisfied	1 (2.5)	3 (7.5)		
Dissatisfied	23 (57.5)	17 (42.5)		
Satisfied	15 (37.5)	18 (45.0)		
Very satisfied	1 (2.5)	2 (5.0)		

a Mann-Whitney U test.

b Fisher’s exact test.

### Effect of Multicomponent Exercise on Outcomes

During the exercise program, all participants’ sRPE scores consistently ranged from 5 to 6. The target heart rate range at 60% HRmax was 88 to 98 bpm. The actual HRmax during the sessions ranged from 94 to 164 bpm, reflecting an average intensity achievement of 102% to 167%.

The preintervention and postintervention changes in physical performance, sleep quality, and physiological indicators among the 36 participants in the per-protocol analysis are shown in Table 2. In the per-protocol analysis, statistically significant differences were observed in 3 physical performance measurements: gait speed (Z = −3.282, *P* = .001), 30-second arm curl (Z = −4.727, *P* < .001), and grip strength (Z = −3.239, *P* = .001). Sleep quality, assessed by the CPSQI, also showed a statistically significant difference, with a postintervention median global score of 5.5 (ranging from 0 to 11). Compared to the baseline, this change was substantial (Z = −2.619, *P* = .009). Furthermore, two specific domains of the CPSQI, namely subjective sleep quality and sleep disturbances, showed statistically significant differences compared with preintervention scores (Z = –3.376, *P* = .001; Z = –2.829, *P* = .005). The items of sleep disturbances in the CPSQI included sleep interruptions, nocturia, coughing, breathing discomfort, sensations of feeling cold or hot, nightmares, and pain.

**Table 2.** Post-intervention Changes in Outcomes Within the Intervention Group.

Outcome variables	Intervention group		Z	*P*-value
	Pretest	Posttest		
	Median (IQR)	Median (IQR)		
**Per-protocol analysis (n = 36)**				
**Physical performances**				
Gait Speed Test (s)	3.55 (2.16–13.6)	4 (2.21–11.6)	−3.282	.001
Chair Stand Test (s)	8.45 (4–20)	9.43 (5.83–17.4)	−0.496	.620
30-Second Arm Curl Test (times)	22 (13–32)	25 (14–35)	−4.727	<.001
Grip Strength Test (Kg)	23 (14–41)	26 (12–44)	−3.239	.001
Side-by-Side Stand Test (s)	10 (10–10)	10 (6–10)	−1.000	.317
Tandem Stand Test	10 (6–10)	10 (10–10)	−1.000	.317
2.44-Meter Timed Walk Test (s)	6.92 (4.45–17.3)	7.22 (4.91–17.84)	−1.009	.313
**Sleep quality**				
The CPSQI global scores (point)	7 (3–12)	5.5 (0–11)	−2.619	.009
**Physiological indicator**				
HRR (bpm)	14 (3–32)	19 (2–39)	−2.097	.036
**ITT analysis (N = 40)**				
**Physical performances**				
Gait Speed Test (s)	3.39 (2.16–13.6)	3.91 (2.21–11.6)	−3.246	.001
Chair Stand Test (s)	8.4 (4–20)	9.43 (5.83–17.4)	−1.886	.059
30-Second Arm Curl Test (times)	23 (13–32)	25 (12–44)	−4.669	<.001
Grip Strength Test (Kg)	23 (14–41)	26 (14–45)	−3.645	<.001
Side-by-Side Stand Test (s)	10 (10–10)	10 (10–10)	−1.000	.317
Tandem Stand Test	10 (6–10)	10 (10–10)	−1.000	.317
2.44-Meter Timed Walk Test (s)	6.7 (4.45–17.3)	7.11 (4.91–17.84)	−1.110	.267
**Sleep quality**				
The CPSQI global scores (point)	8 (3–12)	6 (0–12)	−2.583	.010
**Physiological indicator**				
HRR (bpm)	14 (3–36)	17.5 (2–39)	−2.093	.036

Abbreviations: bpm, beats per minute; CPSQI, the Chinese version of the Pittsburgh Sleep Quality Index; HRR, heart rate recovery.

The per-protocol analysis of the secondary outcome indicated that the median HRR increased from 14 bpm (ranging from 3 to 32 bpm) to 19 bpm (ranging from 2 to 39 bpm), with a statistically significant difference (Z = –2.097, *P* = .036). This change resulted in an average improvement of 31%. The ITT analysis that included all enrolled participants (N = 40) showed findings mostly consistent with the per-protocol analysis.

### Comparison of Primary Outcomes Between Intervention and Control Groups

Baseline analysis confirmed no considerable differences between groups in physical performance or sleep quality, ensuring comparability of the results. Post intervention, statistically significant between-group differences were observed in three physical performance measurements between the intervention group (n = 36) and the control group (n = 39) in the per-protocol analysis, as shown in Table 3. These included the 30-Second Arm Curl (Z = −3.083, *P* = .002), Grip Strength (Z = −2.329, *P* = .02), and Tandem Stand Test (Z = −2.207, *P* = .027). Other performance measurements showed no significant differences with the per-protocol analysis. The CPSQI global score for sleep quality differed statistically significantly between groups post intervention under the per-protocol analysis (Z = −2.036, *P* = .042). In addition, 2 domains of the CPSQI, subjective sleep quality (Z = –3.714, *P* < .001) and sleep disturbances (Z = –2.204, *P* = .028), also demonstrated statistically significant differences. The ITT analysis (N = 80) findings were mostly consistent with the per-protocol analysis.

**Table 3.** Differences in Outcomes Between Intervention and Control Groups.

Outcome variables	Pretest				Posttest			
	Intervention group (IG)	Control group (CG)	Z	*P*-value	Intervention group (IG)	Control group (CG)	Z	*P*-value
	Median (IQR)	Median (IQR)			Median (IQR)	Median (IQR)		
**Per-protocol analysis (IG: n = 36, CG: n = 39)**								
**Physical performances**								
Gait Speed Test (s)	3.55 (2.16–13.6)	4.1 (3–10.9)	−1.930	.054	4 (2.21–11.6)	4.06 (3.08–12)	−0.032	.975
Chair Stand Test (s)	8.45 (4–20)	8.63 (5.11–17.53)	−0.074	.941	9.43 (5.83–17.4)	9.41 (5.1–17.7)	−0.695	.487
30-Second Arm Curl Test (times)	22 (13–32)	21 (10–28)	−1.339	.180	25 (14–35)	21 (11–28)	−3.083	.002
Grip Strength Test (Kg)	23 (14–41)	22 (12–42)	−1.041	.298	26 (12–44)	24 (16–40)	−2.329	.020
Side-by-Side Stand Test (s)	10 (10–10)	10 (10–10)	0.000	−1.000	10 (6–10)	10 (10–10)	−1.041	.298
Tandem Stand Test (s)	10 (6–10)	10 (6–10)	−1.544	.123	10 (10–10)	10 (6–10)	−2.207	.027
2.44-Meter Timed Walk Test (s)	6.92 (4.45–17.3)	7.03 (4.31–20)	0.000	−1.000	7.22 (4.91–17.84)	7.75 (4–30)	−0.408	.683
**Sleep quality**								
The CPSQI global scores (point)	7 (3–12)	7 (4–13)	−0.113	.910	5.5 (0–11)	7 (4–14)	−2.036	.042
**Intention-to-treat analysis (IG: N = 40, CG: N = 40)**								
**Physical performances**								
Gait Speed Test (s)	3.39 (2.16–13.6)	4.06 (3–10.9)	−1.829	.067	3.91 (2.21–11.6)	4.07 (3.08–12)	−0.308	.758
Chair Stand Test (s)	8.4 (4–20)	8.51 (5.11–17.53)	−0.346	.729	9.43 (5.83–17.4)	9.36 (5.1–17.7)	−0.736	.462
30-Second Arm Curl Test (times)	23 (13–32)	21 (10–28)	−1.755	.079	25 (12–44)	21 (11–28)	−3.386	<.001
Grip Strength Test (Kg)	23 (14–41)	22 (12–42)	−0.589	.556	26 (14–45)	24 (16–40)	−2.640	.008
Side-by-Side Stand Test (s)	10 (10–10)	10 (10–10)	0.000	−1.000	10 (6–10)	10 (10–10)	−1.000	.317
Tandem Stand test (s)	10 (6–10)	10 (6–10)	−1.644	.100	10 (10–10)	10 (6–10)	−2.293	.022
2.44-Meter Timed Walk Test (s)	6.7 (4.45–17.3)	7.12 (4.31–20)	−0.553	.580	7.11 (4.91–17.84)	7.77 (4–30)	−0.919	.358
**Sleep quality**								
The CPSQI global scores (point)	8 (3–12)	7 (4–14)	−1.487	.137	6 (0–12)	7 (4–14)	−2.080	.048

Abbreviation: CPSQI, the Chinese Pittsburgh Sleep Quality Index.

## Discussion

This study evaluated the impact of a structured, multicomponent exercise program on physical performance and sleep quality in community-dwelling older adults. Following the intervention, positive changes were observed in gait speed, arm curl repetitions, grip strength, and CPSQI global scores. Participants in the intervention group demonstrated significant improvements in muscular strength and endurance after completing the 8-week program, aligning with previous findings.^9^ Similarly, HRR values improved in the intervention group post training in this study, consistent with earlier studies that indicated exercise resulted in HRR improvement in healthy older adults.^25^ However, no significant changes in balance were observed in this study, potentially due to participants’ high baseline balance levels.

The improvement in sleep disturbance observed in this study is consistent with other studies, which suggest that moderate-intensity exercise improves sleep quality.^26^ Despite these gains, posttest CPSQI scores remained above 5, indicating persistent sleep issues; some participants reported that this related to nocturia and joint pain. Following the intervention in this study, a short-term reduction in sleep disturbances was observed. Because longer exercise training improved sleep more significantly,^26^ further research is needed to evaluate the sustained effects of multicomponent exercise programs on sleep in older adults.

Considerable evidence supports the notion that regular physical activity contributes to enhanced psychological well-being,^4^ a finding that is also echoed by the study participants’ positive feedback. Some participants reported their mental focus and mood were improved, with comments such as, “I felt most focused during exercise” and “I felt much better emotionally.” The intervention in this study followed the guidelines of the ACSM and was designed based on aerobic and resistance training protocols from previous studies. However, this program added some additional elements to enhance participant motivation and persistence. For example, it encouraged social interaction during the extremity stretching exercises in the warm-up session, where participants gave each other high-fives while the coach provided timely positive verbal praise. The monitoring device’s visual image display panel was connected to the isokinetic training devices, helping participants understand their improvement through visual feedback. For example, exercise intensity was represented by a pie chart on the isokinetic training device. When a participant reached the required exercise intensity, the pie chart was displayed in green; at 60% of the required intensity, it appeared orange; and below 30%, it turned red. Each participant had a unique identification number, which they used to log in before exercising, allowing the system to record changes in heart rate and exercise duration for each session. These positive experiences were contributing factors to continuing to join the exercise program. The intervention used in this study served as a practical reference for developing community-based exercise programs in older adults. The study found that common enabling factors of regular exercise included social support, environment suitability, and motivation.^27,28^ In lower-middle-income countries, community-based health-promotion programs could be feasible and applicable for improving mental health in older adults.^27^ This study overcame a common transportation barrier, achieving high adherence, by collaborating with community care sites and providing a shuttle bus service. To support sustainable implementation in the community, governments could further strengthen their health-promotion policies by implementing enabling factors such as motivation. For example, citizens could earn bonus points by attending sports venues or joining exercise classes, which could then be used to offset fees.

The overall compliance rate was high in this study, which may reflect that participants from community care sites recognized the benefits of continuous exercise in maintaining their health, or most of the participants in this study were female and that the design of exercise activities was more in line with women’s preferences, thereby leading to continued participation in exercise interventions. On the other hand, older males may be influenced by the gender stereotype that accepting guidance means not being strong enough or independent, which may reduce their willingness to participate in such activities. Therefore, it is essential to design group exercise programs that incorporate cultural sensitivity to increase the participation of older adults of all genders in these programs.^27,29^ It is suggested that future group exercise programs incorporate more dynamic and challenging elements for older men, such as boxing postures and interesting competitions. For example, aerobic step training could be combined with pedometers for group challenges, or the type of music used during exercise could be adjusted according to the participants’ interests. In addition, before the group exercise begins, co-creating the exercise program with the specific group of older adults could also help increase their motivation and sense of belonging.

Although this study successfully addressed its primary research objective, it relied on a self-report scale to evaluate sleep quality, which might have resulted in recall bias or socially desirable responding. In addition, the quasi-experimental design without randomization may have introduced potential selection bias, although the baseline characteristics between the two groups showed no significant differences. The exercise dosage of the intervention in this study did not fully conform to the ACSM exercise guidelines because the program’s schedule was adjusted based on responses from participants and stakeholders to better meet their demands. Despite these adjustments, participants continued to demonstrate significant differences in the partial assessment of muscular strength and endurance. These outcomes may be attributed to the optimization of the tailoring of interventions to increase engagement in participants.^30^

## Supplemental Material

sj-doc-1-aph-10.1177_10105395261469631 – Supplemental material for The Effects of a Multicomponent Group Exercise Program on Physical Performance and Sleep Quality in Older Adults in Taiwan Supplemental material, sj-doc-1-aph-10.1177_10105395261469631 for The Effects of a Multicomponent Group Exercise Program on Physical Performance and Sleep Quality in Older Adults in Taiwan by Yi-Ling Tseng, Kuei-Min Chen, Min-Huan Wu, Jeng Liu, Ying-Chi Liang and Wendy Moyle in Asia Pacific Journal of Public Health
