# Supplementary material for: The Effects of a Multicomponent Group Exercise Program on Physical Performance and Sleep Quality in Older Adults in Taiwan
Source: Asia Pac J Public Health. 2026 Jul 25;38(6-7):474–82. doi: 10.1177/10105395261469631 (PMC13598308; doi:10.1177/10105395261469631)
Supplement: Supplementary file 1 [file sj-doc-1-aph-10.1177_10105395261469631.doc]

**Supplement Materials**

**Supplement 1. Flowchart for Data Collection in the Multi-component Exercise Program**

**Intervention.**

Participants were recruited (n=80)

Intervention group (n=40)

Data analysis (PP: n = 75; ITT: N = 80)

Pre-test assessment (n=40)

Control group (n=40)

Pre-test assessment (n=40)

Post-test assessment (n=36)

Attrition reasons:

• Hospitalization (n=2)

• Attendance rate < 80% (n=2)

Post-test assessment (n=39)

Attrition reasons:

• Hospitalization (n=1)

• Received an 8-week exercise program at a senior fitness club alongside their regular community activities

• Continued regular community activities

Allocation

Enrollment

Assessed for eligibility at four long-term care stations (n=127)

Excluded reasons:

• Age ineligibility (n=6)

• Contraindications to exercise (n=4)

• Required ambulation aids (n=25)

• Refused to participate (n=12)

**Supplement 2. Details of the intervention content and schedule.**

| Exercise Modalities | Exercise Type | Frequency | Intensity | Duration | Functional  Fitness Items |
| --- | --- | --- | --- | --- | --- |
| Warm-Up Exercises | a. Extremity Stretching Exercises:  - Seated Chest Expansion Stretch.  - Seated shoulder abduction Stretch.  - Seated Knee Extension Stretch.  - Seated Adductor Stretch.  b. Flexibility Exercises:  - Seated Hip Flexion Stretch.  - Seated Trunk Rotation Stretch. | - Each cycle included these two exercises.  - Three cycles each time, once per week. | - Using a resistance band (medium resistance).  - Stretch to the point of maximum tolerable tension, holding the stretch for 10 seconds. | 10  minutes | -Muscular strength. and Flexibility |
| Aerobic Exercises | a. Whole Body Vibration. | - Three cycles each time, once per week. | - Vibration frequency: 20-35 Hz.  - Vibration magnitude: 2-5 mm.  - A vertical, side-alternating platform. | 15  minutes | -Muscular strength.  -Balance. |
|  | b. Treadmill or Stationary Bicycle. | - One cycle each time, once per week. | - Reached 60% of HRmax.  - Target sRPE: 5-6. | 30  minutes | -Cardiorespiratory endurance. |
| Breaking time | | | | 5 minutes |  |
| Resistance Exercises | a. Isokinetic training devices:  Chest Press, Rowing, Incline Shoulder Press, Hip Adduction/Abduction, Leg Curl/Extension, and Leg Press Training. | - Each cycle comprised five training exercises, with each exercise performed for 15 repetitions over four sets.  - Three cycles were performed once per week. | - Reached 60% of HRmax.  - Target sRPE: 5-6.  - Initial resistance intensity was set at 40% 1RM and adjusted to 50–60% based on individual adaptation. | 35  minutes | -Muscular strength.  -Cardiorespiratory   endurance. |
| Cool Down Exercises | a. Slow Walking. | - Once per week. |  | 5  minutes |  |
